# Predictors of Hospitalization in Breakthrough COVID-19 among Fully Vaccinated Individuals with Immune-Mediated Rheumatic Diseases: Data from SAFER-Study

**DOI:** 10.3390/vaccines12091031

**Published:** 2024-09-09

**Authors:** Débora Cerqueira Calderaro, Valéria Valim, Gilda Aparecida Ferreira, Ketty Lysie Libardi Lira Machado, Priscila Dias Cardoso Ribeiro, Sandra Lúcia Euzébio Ribeiro, Natalia Sarzi Sartori, Rodrigo Poubel Vieira de Rezende, Ana Karla Guedes de Melo, Vitor Alves Cruz, Adah Sophia Rodrigues Vieira, Adriana Maria Kakehasi, Aline Teixeira de Landa, Ana Paula Neves Burian, Flávia Maria Matos Melo Campos Peixoto, Camila Maria Paiva França Telles, Rafaela Cavalheiro do Espírito Santo, Katia Lino Baptista, Yasmin Gurtler Pinheiro de Oliveira, Vanessa de Oliveira Magalhães, Raquel Lima de Lima, Erika Biegelmeyer, Pietra Zava Lorencini, Andréa Teixeira-Carvalho, Edgard Torres dos Reis-Neto, Emília Inoue Sato, Marcelo de Medeiros Pinheiro, Odirlei André Monticielo, Viviane Angelina de Souza, Ricardo Machado Xavier, Gecilmara Salviato Pileggi

**Affiliations:** 1 Locomotor System Department, Faculdade de Medicina, Universidade Federal de Minas Gerais (UFMG), Belo Horizonte 30130-100, MG, Brazil; gildaferreira9@gmail.com (G.A.F.);; 2 Hospital Universitário Cassiano Antônio Moraes (HUCAM), Universidade Federal do Espírito Santo (UFES), Vitória 29041-295, ES, Brazil; val.valim@gmail.com (V.V.); drakettymachado@gmail.com (K.L.L.L.M.); yasmingurtler@hotmail.com (Y.G.P.d.O.); pietra.lorencini@edu.ufes.br (P.Z.L.); 3 Escola Paulista de Medicina (EPM), Universidade Federal de São Paulo (UNIFESP), São Paulo 04023-062, SP, Brazil; pri.dcr@gmail.com (P.D.C.R.); flaviacampospeixoto@gmail.com (F.M.M.M.C.P.); vanessa.reumato@gmail.com (V.d.O.M.); erika.biegel@gmail.com (E.B.); edgard.torres@unifesp.br (E.T.d.R.-N.); eisato@unifesp.br (E.I.S.); mpinheiro@uol.com.br (M.d.M.P.); gecilmara@gmail.com (G.S.P.); 4 Escola de Medicina, Universidade Federal do Amazonas (UFAM), Manaus 69067-005, AM, Brazil; sandraler04@gmail.com (S.L.E.R.); camilapaiva2003@hotmail.com (C.M.P.F.T.); raquel.ieq@gmail.com (R.L.d.L.); 5 Serviço de Reumatologia, Hospital de Clínicas de Porto Alegre, Universidade Federal do Rio Grande do Sul (UFRGS), Porto Alegre 90010-150, RS, Brazil; nataliasartori2007@yahoo.com.br (N.S.S.); rafaela.esef@gmail.com (R.C.d.E.S.); omonticielo@gmail.com (O.A.M.); rxavier10@gmail.com (R.M.X.); 6 Universidade Federal Fluminense (UFF), Rio de Janeiro 24020-140, RJ, Brazil; ropoubel@id.uff.br (R.P.V.d.R.); linokatia@gmail.com (K.L.B.); 7 Hospital Universitário Lauro Wanderley, Universidade Federal da Paraíba (UFPB), João Pessoa 58051-900, PB, Brazil; anakarlagmelo@gmail.com; 8 Faculdade de Medicina, Universidade Federal de Goiás (UFG), Goiânia 74690-900, GO, Brazil; vitorcruz@ufg.br; 9 Hospital Geral de Fortaleza (HGF), Universidade de Fortaleza (UNIFOR), Fortaleza 60150-160, CE, Brazil; adahsophia0820@gmail.com; 10 Faculdade de Medicina, Universidade Federal de Juiz de Fora, Juiz de Fora 36036-900, MG, Brazil; aalineland@yahoo.com.br (A.T.d.L.); vivi.reumato@gmail.com (V.A.d.S.); 11 Centro de Referências para Imunobiológicos Especiais (CRIE), Secretaria de Saúde do Estado do Espírito Santo, Vitória 29050-360, ES, Brazil; anapaulaburian@gmail.com; 12 Health Research and Innovation Science Centre, Klaipeda University, 92294 Klaipeda, Lithuania; 13 Instituto Renè Rachou, Fundação Oswaldo Cruz (FIOCRUZ-Minas), Belo Horizonte 30190-002, MG, Brazil; andrea.teixeira@fiocruz.br

**Keywords:** COVID-19, COVID-19 vaccines, health disparities, breakthrough COVID-19 infections, rheumatic diseases, vaccination of immune compromised patients, COVID-19 vaccine booster shot

## Abstract

Breakthrough COVID-19 (occurring in fully vaccinated people) has been described. Data on its characteristics among immune-mediated rheumatic disease (IMRD) patients are scarce. This study describes breakthrough COVID-19 occurring in IMRD patients participating in the SAFER-study, a Brazilian multicentric cohort evaluating the safety, effectiveness, and immunogenicity of SARS-CoV-2 vaccines in patients with autoimmune diseases. A descriptive analysis of the population and a binary logistic regression model were performed to evaluate the predictors of COVID-19-related hospitalization. A *p*-value < 0.05 was significant. The included 160 patients were predominantly females (83.1%), with a mean (SD) age of 40.23 (13.19) years. The patients received two (19%), three (70%), or four (11%) vaccine doses. The initial two-dose series was mainly with ChAdOx1 (Oxford/AstraZeneca) (58%) or BBIBP-CorV (Sinopharm-Beijing) (34%). The first booster (n = 150) was with BNT162b2 (BioNtech/Fosun Pharma/Pfizer) (63%) or ChAdOx1 (29%). The second booster (n = 112) was with BNT162b2 (40%) or ChAdOx1 (26%). The COVID-19 hospitalization rate was 17.5%. IMRD moderate/high activity (OR: 5.84; CI: 1.9–18.5; *p* = 0.002) and treatment with corticosteroids (OR: 2.94; CI: 1.02–8.49; *p* = 0.0043) were associated with higher odds of hospitalization, while increasing the number of vaccine doses was protective (OR: 0.37; CI: 0.15–0.9; *p* = 0.032). These findings, along with previous reassuring results about the safety of the COVID-19 vaccines, argue in favor of booster vaccination in IMRD patients.

## 1. Introduction

The coronavirus disease 2019 (COVID-19) pandemic has been a serious cause for concern among patients with immune-mediated inflammatory and rheumatic diseases (IMID and IMRD). These patients are a unique vulnerable subgroup, as they typically require long-term treatment with multiple immunosuppressive and immunomodulatory (IS/IM) therapy, which is associated with impaired host response to infections and vaccination. In addition, these patients may also have multiple sequelae of their disease (e.g., impaired strength, lung fibrosis, chronic kidney disease) and frequent comorbidities (e.g., cardiovascular disease, obesity, and diabetes). Thus, this patient subgroup may be at greater risk of contracting SARS-CoV-2 infection and severe COVID-19 complications compared to healthy controls [1].

Data from the registries from Argentina, Mexico, and Brazil, including more than 4000 adult IMID patients with COVID-19, reported a hospitalization rate of 22.7% and a mortality rate of 5.3%. Although these registries did not include a control group, the general population mortality from COVID-19 in these countries was nearly 50% lower than that found in this study [2].

In patients with IMID, SARS-CoV-2 vaccines is well tolerated, and it is not associated with a significant increase in disease activity; thus, current evidence suggests that the benefits of vaccination outweigh the potential risks of adverse effects [3].

A major concern is the possibility of attenuated immunogenicity and, consequently, reduced efficacy of vaccines induced by the concomitant use of IS/IM therapies in these patients, which could leave them vulnerable to breakthrough COVID-19 infections [4,5,6]. A systematic review highlighted the risk of low immunogenicity of COVID-19 vaccines in immunocompromised populations. It included 31 studies reporting immunogenicity in 4680 patients with IMID. Non-response rates (antibody and/or cellular responses) ranged from 0% to 63%. Specific treatments for the inflammatory disease that negatively impacted post-vaccine response included B-cell depleting agents, methotrexate, and other disease-modifying anti-rheumatic drugs (DMARDs) or corticosteroids [7].

Some evidence, indeed, supports the recommendation to administer a third dose of COVID-19 vaccine in patients with IMID, since, in this group, double and triple-vaccinated patients showed a lower rate of COVID-19-related hospitalization and triple-vaccinated patients presented a reduction in COVID-19-related deaths [3].

Data on the presentation and the severity of breakthrough infections in patients with IMRD vaccinated against COVID-19 in Brazil are lacking.

This study presents an analysis of the SARS-CoV-2 breakthrough infections among fully vaccinated individuals with immune-mediated rheumatic diseases from the “Study of safety, effectiveness and immunogenicity duration after vaccination against the new SARS-CoV-2 in patients with immune-mediated inflammatory diseases (SAFER-Study)”, of the Brazilian Society of Rheumatology. It intends to describe the profile and the severity of SARS-CoV-2 infection in fully vaccinated IMRD patients presenting breakthrough COVID-19, and the predictors of hospitalization among these patients.

## 2. Materials and Methods

### 2.1. SAFER-Study

The “Study of safety, effectiveness and immunogenicity duration after vaccination against the new SARS-CoV-2 in patients with immune-mediated inflammatory diseases (SAFER-Study)” is a Brazilian national, multicenter, observational, longitudinal real-world cohort of consecutive patients with IMRD or IMID who have been vaccinated for SARS-CoV-2, evaluating the safety, effectiveness, and immunogenicity of SARS-CoV-2 vaccines in this population. The inclusion period was from June/2021 to March/2024. Follow-up is still ongoing. The present study presents data on the occurrence of breakthrough COVID-19 among patients participating in the SAFER-study. Data from June/2021 to February/2023 were included.

The SAFER-study included pediatric or adult patients, with a prior diagnosis of IMID or IMRD. IMRD diagnosis, according to the American College of Rheumatology (ACR) or the European League against Rheumatism (EULAR) classification criteria, were rheumatoid arthritis, juvenile idiopathic arthritis, spondylarthritis and other inflammatory joint diseases, and systemic lupus erythematosus, as well as Sjögren’s disease, inflammatory myopathies, systemic vasculitis, systemic sclerosis, mixed connective tissue disease, and other connective tissue diseases or other rheumatic diseases. IMID patients participating in the SAFER-study were those diagnosed with inflammatory bowel disease (IBD) or psoriasis. All patients gave written informed consent. The SAFER-study is a non-probability sampling study with enrollment of consecutive patients who met the selection criteria.

All patients included in this study received one to four doses of a SARS-CoV-2 vaccine. The vaccines available in Brazil during the data assessment for the breakthrough COVID-19 infection were the CoronaVac vaccine (Sinovac Biotech), the AstraZeneca vaccine (ChAdOx1), and the vaccines developed by Pfizer (BNT162b2) and Janssen (Ad26.COV2.S). The SAFER-study patients will be followed up from the inclusion until December of 2024.

Baseline evaluation took place before the first dose of SARS-CoV-2 vaccine application. Follow-up visits occurred 4 weeks after each vaccine dose and, later, every 3 months, until 12 months of complete follow-up.

Sociodemographic data, the presence of comorbidities, characteristics, and severity of IMRD or IMID, treatments received, and clinical characteristics and outcomes of SARS-CoV-2 infection were recorded, whether previous or acquired during the study.

In addition, the date and place of vaccination, type of vaccine applied, scheme, and indication were registered. Blood samples were collected for immunogenicity analysis. Adverse events, disease flares, and new immune-mediated manifestations related to the vaccines were registered.

### 2.2. Recruitment of Patients for Participation in This Study

IMID patients who are regularly followed-up in SAFER-study participating centers, and have provided information on COVID-19 vaccination, were consecutively invited to participate in this study by their attending physicians during their medical appointment, scheduled in a real-life setting. The participants were not remunerated but received reimbursement for travel and food expenses related to the follow-up visits for this study. Information on the number of patients refusing to participate, or the reasons for declining, was not recorded.

### 2.3. Breakthrough COVID-19 Definition

Breakthrough infection among fully vaccinated individuals was defined according to the US Centers for Disease Control and Prevention (CDC) as infection occurring ≥ 14 days after the second dose in a two-dose series or ≥14 days after a single-dose vaccine [8].

### 2.4. COVID-19 Diagnosis

COVID-19 was confirmed by a positive reverse transcriptase-polymerase chain reaction (RT-PCR) or antigen (Ag) test for the SARS-CoV-2 virus obtained from a nasopharyngeal or oropharyngeal swab.

### 2.5. Outcomes

The primary outcome of this study was the clinical presentation of breakthrough COVID-19. The secondary outcomes included severe COVID-19 related events, such as the need of supplemental oxygen or ventilatory assistance, hospitalization, infectious complications, and death.

### 2.6. Inclusion and Exclusion Criteria

Adult patients, fully vaccinated against SARS-CoV-2, with a diagnosis of an IMRD, who developed COVID-19, confirmed by RT-PCR or antigen tests, were included. Patients reporting COVID-19 before the inclusion in the SAFER-study or those who were partially vaccinated (defined as after the first dose or ≤14 days following the second dose in a two-dose series, or of a single-dose vaccine), those with the diagnosis of IBD or psoriasis (IMID), and patients who did not present COVID-19 were excluded. Patients with chronic infections (e.g., HIV infection, chronic viral B or C-hepatitis) or neoplasms as comorbidities were also excluded.

### 2.7. Study Variables

Demographic variables including age, sex, and self-declared color were recorded. The presence and the number of comorbidities (including cardiomyopathy, diabetes mellitus, pulmonary disease, chronic kidney disease, systemic arterial hypertension and obesity) were also noted. Information regarding IMRD diagnosis, treatment, and disease activity at the time of SARS-CoV-2 vaccination and infection was documented.

Immune-mediated inflammatory diseases differ regarding the DMARDs approved for their treatment. To minimize the impact of this heterogeneity on the associations of interest, diagnostic categories were defined according to the approach established by the COVID-19 Global Rheumatology Alliance (C19-GRA): inflammatory joint diseases (IJDs), connective tissue diseases (CTDs)/vasculitis, and other rheumatic diseases. In addition, treatments were categorized as follows: glucocorticoids, conventional DMARDs (cDMARDs; antimalarials, leflunomide, methotrexate, sulfasalazine), immunosuppressants (IS; azathioprine, cyclophosphamide, cyclosporine, mycophenolate mofetil/mycophenolic acid, tacrolimus), tumor necrosis factor inhibitors (TNFI), Rituximab, biologic DMARDs (bDMARDs; abatacept, belimumab, interleukin 1 [IL-1], IL-6, IL-12/23, IL-17, and IL-23 inhibitors), targeted synthetic DMARDs (tsDMARDs; Janus kinase inhibitors [JAKi]) [9].

IMRD activity was stratified into categories according to the treating physician’s assessment and grouped as remission/low or moderate/high disease activity.

Regarding SARS-CoV-2 vaccination, the date and type of vaccine received (CoronaVac vaccine [Sinovac Biotech], the AstraZeneca vaccine [ChAdOx1], and the vaccines developed by Pfizer [BNT162b2] and Janssen [Ad26.COV2.S]) were recorded. The completion of the initial two-dose or single-dose regimen and the receipt of one to two booster doses were registered.

Breakthrough COVID-19 clinical presentation (including asymptomatic infection, cough, dyspnea, sore throat, fever, dysgeusia, hyposmia, myalgia, malaise, headache, and rhinorrhea), date of diagnosis, confirmatory test performed, and outcomes of interest (hospitalization, respiratory assistance, infectious complications, and death) were recorded.

### 2.8. Data Management and Monitoring

Data were collected through face-to-face interviews, virtual consultations, or telephone calls, in addition to the review of medical records, according to availability. Data were entered into the Research Electronic Data Capture Platform (Redcap, https://redcap.reumatologia.org.br/, accessed on 30 March 2023). The study is ongoing. Datasets of this study are not deposited in a publicly available database study. Data will be available upon reasonable request.

### 2.9. Ethical Considerations

The study protocol and informed consent forms were approved by the ethics committee at the coordinating center (CAAE: 43479221.0.1001.5505) and from the independent ethics committee at all participating centers. All patients voluntarily agreeing to participate signed the informed consent prior to enrollment and data collection.

This study was conducted in accordance with the Good Clinical Practice (GCP) guidelines, the International Conference on Harmonization (ICH) standards, and the ethical principles established in the Declaration of Helsinki, local laws (law 3301/09), and the regulations of the local ethics committee.

Personal identification data were kept anonymous and protected according to international and national regulations to ensure confidentiality, in accordance with the Law on Protection of Personal Data.

For the purposes of this project, only medical researchers had access to the patients’ medical records to obtain the data required for the investigation, thus ensuring their confidentiality.

### 2.10. Statistical Analysis

Descriptive analyses of sociodemographic and clinical data were conducted. Continuous variables are presented as mean and standard deviation if normally distributed, or as median and interquartile range otherwise. Categorical variables are presented as frequencies and percentages.

Comparative analyses of patients requiring hospitalization due to COVID-19 versus those not requiring hospitalization employed Student’s t-test or the Mann–Whitney test for continuous variables, according to a normal or non-normal distribution. Categorial variables were compared using the Chi-square or Fisher’s exact test, as appropriate.

A binary logistic multiple regression, applying a backward stepwise selection method was performed to evaluate the variables associated with breakthrough COVID-19-related hospitalization. Variables presenting a *p*-value ≤ 0.2 were included in the initial model and were sequentially excluded until only significant variables remained in the final model. Patients with missing data regarding the variables of interest were excluded from the comparative and the regression analyses. A *p*-value < 0.05 was deemed statistically significant. Statistical analyses and model development were performed with R version 4.3.1 [10] with the software RStudio version 2023.6 [11], along with the packages “tidyverse” [12] and “gtsummary” [13].

## 3. Results

### 3.1. Study Population

**Figure 1:**
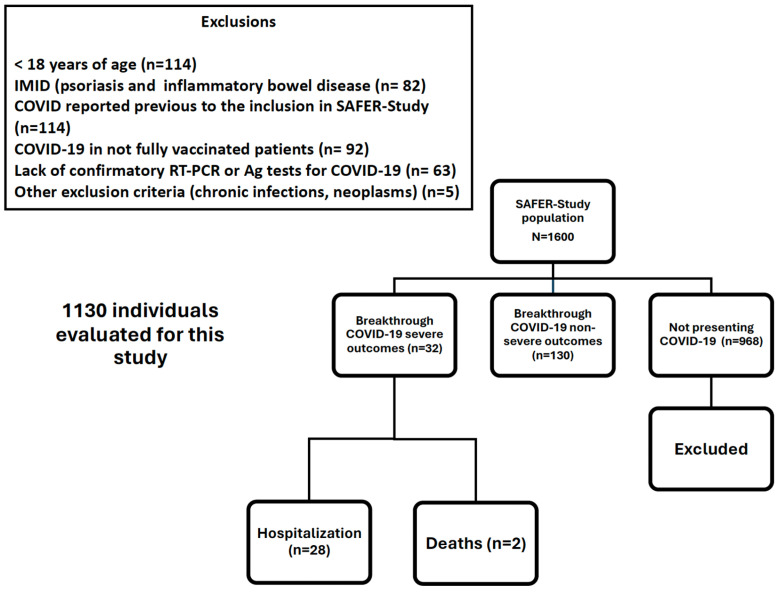
Patient selection for the SAFER-study.

By the time the data for this analysis were extracted, the SAFER-study had included 1600 IMID patients. In this analysis, 162 cases of breakthrough COVID-19 reported in the study database are described. Figure 1 depicts the algorithm used for patient selection in this study.

In the SAFER-study database, 162 cases of breakthrough COVID-19 were described: 132 presented non-severe outcomes, 28 required hospitalization, and two patients died.

The characteristics of the 160 patients surviving breakthrough COVID-19, along with the comparative analysis between hospitalized (n = 28, 17.5%) and non-hospitalized patients (n = 132, 82.5%), are presented in Table 1.

Age, gender distribution, and prevalence of various rheumatic diseases and comorbidities were similar between hospitalized and non-hospitalized patients. The hospitalized patients were more likely to use glucocorticoids (34% vs. 10%) and exhibit moderate-to-high IMRD activity (37.5% vs. 11.5%).

### 3.2. Vaccination

Thirty-one (19.4%) patients received only the two-dose regimen of vaccines, while 112 (70%) and 17 (10.6%) received one and two booster doses, respectively. The non-hospitalized group received a higher number of vaccine doses (*p* = 0.028).

The initial two-dose regimen primarily utilized the AstraZeneca vaccine (ChAdOx1) in 93 (58.1%) patients, followed by the CoronaVac vaccine (Sinovac Biotech) in 53 (34.4%), and the Pfizer (BNT162b2) vaccine in 12 (7.5%). The first booster dose was predominantly with the Pfizer (BNT162b2) vaccine, administered to 101 (63.1%) patients, followed by the AstraZeneca vaccine (ChAdOx1) [n = 46 (28.7%)] and the CoronaVac vaccine (Sinovac Biotech) [n = 3 (1.9%)]. The second booster dose again presented a predominance of the Pfizer (BNT162b2) vaccine [n = 64 (40%)], followed by the AstraZeneca vaccine (ChAdOx1) [n = 41 (25.6%)] and Janssen (Ad26.COV2.S) [n = 7 (4.4%)]. The multiple vaccination schemes employed did not correlate with hospitalization rates.

Prior to vaccination, patients were using oral glucocorticoids (n = 28), cDMARDs (n = 133), bDMARDs (n = 41), Rituximab (n = 13), immunosuppressants (n = 35), and tsDMARDs (n = 2). These frequencies were similar between non-hospitalized and hospitalized individuals.

### 3.3. Breakthrough COVID-19 Presentation

Breakthrough COVID-19 occurred in a median of 113.46 days (IQR: 75.25–134.75) after the last vaccine dose, with no significant difference between non-hospitalized and hospitalized patients (median 109 vs. 100 days, *p* = 0.3).

Confirmatory tests were RT-PCR in 73 (46%) and antigen test in 77 (48%) individuals. By the time of the COVID-19 diagnosis, 47 (29%) patients were not receiving any antirheumatic medications.

Most (96%) patients reported COVID-19 symptoms, including headache (57%), cough (54%), rhinorrhea (51%), sore throat (49%), fever (46%), fatigue (32%), asthenia (31%) hyposmia (20%), dysgeusia (18%), and dyspnea (18%), diarrhea (17%), nausea (14%), dizziness (9%), vomiting (4%), myalgia (3%), arthralgias (3%). COVID-19 symptoms of cough (51% vs. 71%, *p* = 0.046), dyspnea (39% vs. 13%, *p* = 0.001), dysgeusia (36% vs. 18%, *p* = 0.03) and fever (42% vs. 64%, *p* = 0.03) were more frequently reported by those who were hospitalized.

The duration of COVID-19 symptoms ranged from 1 to 60 days [median (IQR): 8.95 (5–12). Patients requiring hospitalization had an average in-hospital stay of 1 to 33 [median (IQR): 10.2 (1–23.5)] days. Among the hospitalized patients, six required supplemental oxygen, one required non-invasive ventilation, and five developed infectious complications (bacterial pneumonia) associated with COVID-19.

### 3.4. Determinants of COVID-19-Related Hospitalization

**Figure 2:**
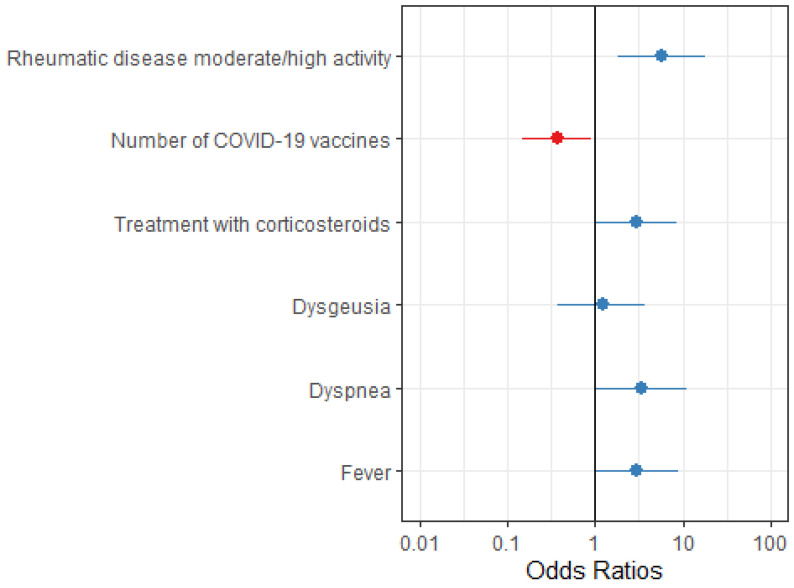
Multivariate analysis of the COVID-19-related hospitalization determinants. Legend for Figure 2: OR, 95%CI for the variables independently associated with breakthrough COVID-19-related hospitalization.

Univariate and multivariate analyses assessing predictors of COVID-19-related hospitalization are summarized in Table 2. In this population, moderate-to-high IMRD activity, corticosteroid use, and presenting with fever or dyspnea were associated with increased odds of hospitalization, while the number of COVID-19 doses correlated with reduced odds of hospitalization (Table 2, Figure 2).

### 3.5. COVID-19-Related Deaths

Three deaths attributed to COVID-19 were recorded in the SAFER-study. Two deaths were attributed to breakthrough COVID-19 (fatality rate: 1.2%) and one occurred in a not fully vaccinated patient (after the first dose of a two-dose regimen). The two breakthrough COVID-19-related deaths were identified retrospectively and are described in Table 3.

## 4. Discussion

This study brings important information on breakthrough COVID-19 clinical presentation and hospitalization determinants in a Brazilian population of IMRD patients. It corroborates previous data on the association of moderate-to-high activity of the IMRD and current treatment with glucocorticoids with severe COVID-19 outcomes and underscores COVID-19 symptoms of fever or dyspnea as predictors of hospitalization associated with this infection. It also suggests a protective effect of the increased number of vaccine doses, highlighting the importance of booster vaccination in these patients.

In this cohort, only two (1.2%) COVID-19 related deaths were reported in fully vaccinated individuals, precluding the authors from the evaluation of the determinants of death in these patients. In the study by the Global Rheumatology Alliance (GRA), reporting breakthrough COVID-19 in 86 fully vaccinated people with RD, five (6%) deaths occurred. The determinants of COVID-19-related deaths were also not evaluated in that study, but four of these five individuals were older than 60 years, and three individuals were on B-cell depleting therapy (BCDT) at the time of vaccination [4]. In the present study, from the two patients who died, one patient was older than 60 years, and none received Rituximab before vaccination; however, both were using glucocorticoids, one of the predictors of hospitalization in our population.

The hospitalization rate for COVID-19 in this study (17.5%) was lower than that reported in a Latin America study (22.7%), in which data on vaccination were not available [2]. Accordingly, better outcomes, with lower mortality and hospitalization rates in partially or fully vaccinated IMRD patients, compared to unvaccinated individuals, have been described [14].

The hospitalization rate described In our study was also lower than that reported by the C19-GRA study, which included COVID-19 in 86 fully vaccinated patients with RD, of whom 22 (26%) were hospitalized and 5 (6%) died [4]. These disparities in hospitalization rates may be attributed to the different vaccination protocols and genetic backgrounds from the different populations from both studies. They may also associate with the higher prevalence of patients receiving booster doses of the vaccine in our study, since boosters have been shown to significantly decrease the frequency of COVID-19-related hospital admissions and deaths in individuals with systemic IMRD [15].

The mean period between the infection and the second vaccine dose, or the first or second booster, and the occurrence of breakthrough COVID-19 aligns with previous studies, as well as with the clinical presentation of COVID-19 [4,5,6].

Despite the established efficacy of COVID-19 vaccines, breakthrough infections can occur in those who are fully vaccinated. In IMRD patients, previous studies have shown that specific classes of medications, e.g., BCDT, bDMARDS, TNFI, immunosuppressants, JAKi, and glucocorticoids, can hamper the humoral response and adversely impact both humoral and T-cell-mediated immune responses. Moreover, the decreased severity of breakthrough COVID-19 after booster doses of the vaccines in individuals with systemic RDs, in combination with the reassuring results about the safety of the vaccines, argues in favor of booster vaccination in patients with IMRDs. Thus, the decrease in hospitalization, attributable to the increased number of vaccine doses, as found by the present work, highlight the need for additional booster doses in this population [4,5,6,15,16].

The association of moderate-to-high IMRD activity with the increased risk of COVID-19-related hospitalization and other severe outcomes may be attributed to the immune dysregulation occurring in patients with active disease [2,17].

During the SARS-CoV-2 pandemic, reduced adherence to treatment regimens among IMRD patients, occurring for several reasons, may have contributed to a higher disease activity, correlating with a heightened risk of severe COVID-19 outcomes, regardless of the protective effect of vaccination [17,18].

To the authors’ knowledge, this is the first Brazilian study describing breakthrough COVID-19 in IMRD patients. The main strength of this study is that the population was extracted from a multicenter cohort, conducted by experienced rheumatologists from tertiary rheumatology services across Brazil. The number of confirmed cases and their in-depth description shall also be highlighted.

However, this study has certain limitations, mainly the lack of a control group of healthy individuals presenting with breakthrough COVID-19. Furthermore, the inclusion of a convenience sample and the lack of a record of patients who declined to participate in this study, including the reasons leading to this decision, may have led to a selection bias. Moreover, the loss of asymptomatic breakthrough infections, because we only included patients who presented for testing, and the exclusion of symptomatic patients that did not perform a diagnostic test, may have underestimated the frequency of breakthrough COVID-19. Still, the low occurrence of COVID-19-related deaths precluded the researchers from performing the evaluation of the predictors of death in these patients. The evaluation of the immunogenicity of COVID-19 vaccines in the population of this study is ongoing and will be featured in future publications to further evaluate the vaccination effectiveness in this population.

## 5. Conclusions

In this large cohort study, consisting of fully vaccinated IMRD patients, higher disease activity, or chronic use of corticosteroids, increased the chance of hospitalization due to COVID-19, while booster vaccinations showed a protective effect. The decreased severity of breakthrough COVID-19 symptoms after booster doses of the vaccines in patients with IMRDs, in combination with the reassuring results about the safety of vaccines, argues in favor of booster vaccination in patients with IMRD. New studies, evaluating the impact of breakthrough COVID-19 and the effects of vaccine booster doses in these patients, are warranted.

## Figures and Tables

**Table 1:** Descriptive and comparative analysis of the overall breakthrough COVID-19 cases included, as well as patients not hospitalized (n = 132) versus hospitalized (n = 28) *.

Characteristic	Overall (N = 160 *) ^1^	Non-Hospitalized (N = 132) ^1^	Hospitalized (N = 28) ^1^	*p*-Value ^2^
Age (years)	44.08 (12.81)	44.21 (13.21)	43.46 (10.93)	0.9
Gender [female]	133 (83.1%)	110 (83.3%)	23 (82.1%)	>0.9
Self-declared white color	97 (60.6%)	77 (58.3%)	20 (71.4%)	0.2
CTD/vasculitis/other RD	96 (60.0%)	78 (60%)	18 (64%)	0.67
IJD	62 (39%)	52 (40%)	10 (36%)	0.67
RD time length (years)	9.50 (4.00–15.00)	10.00 (4.00–15.00)	8.50 (3.00–16.00)	0.5
RD moderate/high activity before breakthrough COVID-19	23 (14%)	14 (11.5%)	9 (37.5%)	**0.001**
Comorbidity (any)	102 (63.8%)	83 (62.9%)	19 (67.9%)	0.6
Number of comorbidities	1 (0–1)	1 (0–1.25)	1 (0–1)	0.3
**Rheumatic disease treatment before COVID-19**				
Glucocorticoids use	34 (21%)	24 (10%)	10 (34%)	**0.04**
Glucocorticoids daily doses (mg/prednisone)	7.5 (5–10)	8.75 (5–10)	5 (5–7.5)	0.5
cDMARDs	96 (60%)	79 (60%)	17 (60%)	0.16
bDMARDs	19 (12%)	15 (11.5%)	4 (15%)	0.4
tsDMARDs	3 (1.9%)	1 (0.8%)	2 (7.1%)	0.08
Rituximab	1 (0.6%)	1 (1%)	0 (0%)	0.6
IS	14 (9%)	14 (11%)	0 (0%)	0.13
**COVID-19 manifestations**				
Headache	91 (57%)	72 (55%)	18 (64%)	0.39
Cough	86 (54%)	66 (51%)	20 (71%)	**0.046**
Rhinorrhea	81 (51%)	65 (50%)	16 (57%)	0.49
Sore throat	78 (49%)	60 (46%)	18 (64%)	0.08
Fever	73 (46%)	55 (42%)	18 (64%)	0.03
Fatigue	51 (32%)	40 (31%)	11 (39%)	0.38)
Asthenia	50 (31%)	38 (29%)	10 (36%)	0.49
Hyposmia	32 (20%)	23 (18%)	9 (32%)	0.08
Dysgeusia	29 (18%)	23 (18%)	10 (36%)	**0.03**
Dyspnea	29 (18%)	17 (13%)	11 (39%)	**0.001**
Diarrhea	27 (17%)	22 (17%)	5 (18%)	0.91
Nausea	22 (14%)	16 (12%)	6 (21%)	0.21
Dizziness	14 (9%)	11 (9%)	4 (14%)	0.31
Vomiting	6 (4%)	5 (4%)	2 (7%)	0.61
Myalgia	5 (3%)	5 (4%)	0 (0%)	0.59
Arthralgias	5 (3%)	3 (2%)	2 (7%)	0.22
**Number of vaccine doses**				
Number of COVID-19 vaccine doses	3 (3–4)	3 (2–4)	3 (2–3)	**0.028**
Two-dose regimen	31 (19.4%)	20 (15%)	11 (39%)	**0.02**
Three doses (one booster)	112 (70%)	96 (74%)	14 (50%)	**0.004**
Four doses (two boosters)	17 (10.6%)	14 (11%)	3 (11%)	1.0
Timepoint between vaccination and breakthrough COVID-19 (days)	113.46 (75.25–134.75)	109 (76–134.75)	100 (70–132.25)	0.3

CTD: connective tissue diseases. RD: rheumatic diseases. IJD inflammatory joint diseases. CTD/vasculitis/other rheumatic diseases: 44 systemic lupus erythematosus, 16 vasculitis, 5 systemic sclerosis, 4 inflammatory myopathy, 20 Sjögren’s disease, 3 mixed connective tissue disease and 6 connective diseases overlapping syndrome. IJD (31 rheumatoid arthritis, 1 juvenile idiopathic arthritis, 24 ankylosing spondylitis, 5 psoriatic arthritis, 1 enteropathic arthritis). Comorbidities were cardiomyopathy (n = 4), diabetes mellitus (n = 8), pulmonary disease (n = 8), chronic kidney disease (n = 3), arterial hypertension (n = 54), obesity (n = 20), other comorbidities (n = 53), smoking (n = 2), alcoholism (n = 9). cDMARDs: conventional DMARDs (Methotrexate, Leflunomide, Sulfasalazine, antimalarials). bDMARDs: biologic DMARDs other than Rituximab (abatacept, TNF inhibitors, belimumab, IL-1, IL-6, IL-12/23, IL-17 and IL-23 inhibitors). tsDMARDs: Janus kinase inhibitors (tofacitinib, baricitinib, upadacitinib). IS: immunosuppressants: azathioprine, cyclosporine, tacrolimus, mycophenolate mofetil/mycophenolic acid, cyclophosphamide. * Detailed data of the two breakthrough COVID-19-related deaths were not available, and these patients were not included in this analysis. ^1^ Mean (SD); median (IQR); n (%). ^2^ Mann–Whitney test; Fisher’s exact test; Pearson’s Chi-squared tests.

**Table 2:** Univariate and multivariate analysis of the COVID-19-related hospitalization determinants *.

	Univariate Analysis			Multivariate Analysis	
Characteristic	N	OR, 95%CI	*p*	OR, 95%CI	*p*
White color	160	1.83, 0.78–4.7	0.2	-	-
Rheumatic disease moderate/high activity	146	4.63, 1.68–12.6	**0.003**	5.73, 1.85–18.3	**0.002**
Number of COVID-19 vaccine doses	160	0.42, 0.19–0.92	**0.003**	0.38, 0.15–0.91	**0.035**
Treatment with glucocorticoids before COVID-19	160	2.40, 0.95–5.86	**0.056**	2.92. 1.01–8.45	**0.045**
Lack of treatment with antirheumatic drugs before COVID-19	160	0.46, 0.14–1.2	0.14	-	-
**COVID-19 manifestations**					
Dysgeusia	160	2.58, 1.03–6.27	**0.038**	-	-
Dyspnea	160	4.30, 1.7–10.8	**0.002**	3.44, 1.05–11.2	**0.038**
Sore throat	160	2.10, 0.92–5.06	0.086	-	-
Fever	160	2.45, 1.07–5.92	**0.038**	2.95, 1.05–9.08	**0.047**
Hyposmia	160	2.20, 0.86–5.4	0.090	-	-
Cough	160	2.42, 1.03–6.21	0.051	-	-

OR: odds ratio, CI: confidence interval, *p*: probability. N: number of patients included in the analysis. * Detailed data on the two breakthrough COVID-19-related deaths were not available, and these patients were not included in this analysis.

**Table 3:** Description of the COVID-19-related deaths recorded in SAFER-study.

Characteristic	Case 1	Case 2
Sex	Female	Male
Age (Years)	43	63
IMRD Diagnosis	SLE	IgA-vasculitis
Number of COVID-19 Vaccine Doses	3	3
Dates and Types of COVID-19 Vaccines Administered	Dose 1: CoronaVac (11 September 2021) Dose 2: CoronaVac (13 October 2021) Dose 3: ChAdOx1 (13 October 2022)	Dose 1: ChAdOx1 (5 May 2021) Dose 2: ChAdOx1 (27 May 2021) Dose 3: ChAdOx1 (27 September 2021)
Comorbidities	CKD stage II, arterial hypertension, obesity (BMI: 41)	None
IMRD Treatment	PDN 12.5 mg + MMF	PDN 10 mg
COVID-19 Manifestations	NR	Flu-like symptoms, fever and cough starting on 14 November 2021. Positive COVID-19 on 17 November 2021. Hospitalized on 19 November 2021. He was transferred to the ICU on 23 November 2021, developed SARS, with acute respiratory failure and the need for invasive mechanical ventilation, and AKI. He died on 29 November 2021.
Death Date	19 November 2022	29 November 2021

IMRD: immune-mediated rheumatic disease. SLE: systemic lupus erythematosus. CKD: chronic kidney disease. BMI: body mass index. PDN: prednisone. MMF: mycophenolate mofetil. NR: not reported. SARS: severe acute respiratory syndrome. ICU: intensive care unit. AKI: acute kidney injury.

## Data Availability

The research dataset of this study is not publicly available due to privacy restrictions (they are part of an ongoing study). Requests to access the datasets should be directed to Débora Cerqueira Calderaro (dccalderaro@gmail.com).
